# Nurse-led evidence-based protocolized weaning for invasive mechanical ventilation patients in the ICU: a hybrid type 1 effectiveness-implementation study

**DOI:** 10.1186/s12890-025-03967-5

**Published:** 2025-10-27

**Authors:** Lei Xue, Hongzhan Jiang, Zongyu Wang, Jianan Zhang, Xiaojie Wang, Shuqian Song, Liyun Han, Lianqi Zhang, Yufang Hao, Qinggang Ge

**Affiliations:** 1 Department of Critical Care Medicine, Peking University Third Hospital, Beijing, China; 2 School of Nursing, Beijing University of Chinese Medicine, Beijing, China; 3 Department of Pediatrics, China-Japan Friendship Hospital, Beijing, China; 4 School of Traditional Chinese Medicine, Beijing University of Chinese Medicine, Beijing, China

**Keywords:** Nurse-led weaning, Mechanical ventilation liberation, Implementation science, Intensive care, Evidence-based practice

## Abstract

**Background:**

Prolonged invasive mechanical ventilation (IMV) increases risks of complications and healthcare burdens. Protocol-directed weaning reduces IMV duration but faces implementation barriers. Nurse-led protocolized weaning (NLPW) offers promise, yet evidence on its effectiveness within specific healthcare contexts and systematic implementation processes remains limited.

**Objectives:**

This hybrid type 1 effectiveness-implementation study evaluated the clinical impact and feasibility of implementation of a structured NLPW program in a surgical ICU.

**Methods:**

A mixed-methods design was employed. Quantitative component: A quasi-experimental study with historical controls compared patient outcomes (weaning duration, IMV duration, ICU stay, 24-hours reintubation) before (May-Sep 2023, *n* = 147) and during (May-Aug 2024, *n* = 147) implementing an evidence-based NLPW program guided by the Knowledge-to-Action framework. Qualitative component: Semi-structured interviews informed by the Consolidated Framework for Implementation Research (CFIR) identified implementation barriers and facilitators. Nurses’ knowledge changes and protocol adherence were assessed.

**Results:**

Among 294 included patients (mean age 62.3 ± 17.8 years, 50.3% male), NLPW significantly reduced mean weaning duration (1.66 ± 0.92 vs. 2.8 ± 1.35 h; Δ = 1.2 h, *P* < 0.001) without increasing reintubation rates or shortening total IMV duration. Post-implementation, nurses’ knowledge scores increased significantly (70.3 ± 7.6 to 83.5 ± 5.1; *P* < 0.05), and protocol adherence exceeded 95% for core process indicators. Qualitative analysis revealed three critical success factors: (1) individual behavioral changes, (2) optimization of nursing workflows, and (3) utilization of resources to support clinical practice.

**Conclusions:**

A theory-informed, NLPW program safely accelerated liberation from IMV in surgical ICU patients and enhanced nursing expertise. Successful implementation requires addressing contextual barriers through tailored training, workflow optimization, and resource support. NLPW represents a scalable strategy to standardize ventilator weaning, particularly in settings with limited respiratory therapy support. Future studies should evaluate long-term sustainability and generalizability across diverse ICU populations.

**Patient or public contribution:**

: No Patient or Public Contribution.

**Supplementary Information:**

The online version contains supplementary material available at 10.1186/s12890-025-03967-5.

## Introduction

Invasive mechanical ventilation (IMV) is a cornerstone life-support intervention for critically ill patients experiencing respiratory failure. Globally, an estimated 20 million individuals receive mechanical ventilation annually [1], with approximately 40% of patients admitted to Intensive Care Units (ICUs) requiring IMV [2]. While indispensable, prolonged IMV duration is strongly associated with significant morbidity, including ventilator-associated pneumonia (VAP), ventilator-induced lung injury (VILI), respiratory muscle weakness, gastrointestinal complications (e.g., stress ulcers, bleeding) [3–5], and increased mortality. Furthermore, extended ventilator dependence imposes substantial burdens on healthcare resources and patient costs [6].

Timely liberation from IMV is therefore paramount to mitigate these risks and improve patient outcomes [7, 8]. The process of transitioning patients from full ventilatory support to spontaneous breathing and eventual extubation, known as weaning, constitutes a critical phase [9]. Traditional, empiric weaning, reliant solely on physician judgment and experience, suffers from subjectivity and variability, potentially leading to unnecessary delays or premature attempts, both of which are detrimental [10, 11].

Protocolized weaning, in contrast, employs structured, evidence-based algorithms driven by predefined, objective clinical criteria to systematically assess patient readiness for weaning trials (spontaneous breathing trials - SBTs) and guide the weaning process [12]. Substantial evidence demonstrates that protocolized weaning significantly reduces the duration of mechanical ventilation (by up to 26%) [10], decreases rates of VAP and early reintubation [13], and shortens ICU length of stay compared to non-protocolized approaches [14].

The successful implementation of protocolized weaning hinges on consistent assessment and execution [15]. While physicians, nurses, and respiratory therapists can all perform this role, physician-led protocols may face challenges due to competing clinical demands, potentially delaying assessments and weaning decisions [16, 17]. Respiratory therapists play a vital and effective role [18], but their availability is often limited, especially in regions outside North America [19]. Nurses, by virtue of their continuous presence at the bedside and expertise in monitoring critically ill patients, represent a highly feasible and promising group to lead protocolized weaning initiatives [20]. Growing evidence supports that NLPW can achieve comparable or superior outcomes to physician-led weaning, including reduced ventilation time and ICU stay, without compromising safety, and is well-accepted by physicians [6, 12, 16].

Despite its demonstrated benefits and the strong rationale for nurse-led models, the widespread adoption and consistent implementation of NLPW remain variable and suboptimal [21]. Significant barriers exist, including regional variations in healthcare systems, policy frameworks, professional scopes of practice, resource allocation (e.g., staffing, training), and organizational culture [21–24]. While a robust body of evidence-based guidelines and best practices for weaning exists, effectively translating this knowledge into sustainable, context-specific nursing practice in diverse ICU settings remains a major challenge [25–27].

While NLPW shows promise, robust evidence on its effectiveness within specific healthcare contexts, particularly in China, and a detailed understanding of the implementation processes, barriers, and facilitators required for its successful integration into routine ICU nursing practice, are still needed [28, 29]. Most prior studies focus primarily on clinical outcomes; fewer utilize rigorous implementation science frameworks to guide and evaluate the implementation process itself in the context of NLPW [28, 30, 31].

This hybrid type 1 effectiveness-implementation study aimed to: (1) Evaluate the clinical effectiveness of a structured, evidence-based, NLPW program for adults receiving IMV in a surgical ICU, compared to usual care (historical control). (2) Describe and analyze the implementation process of this NLPW program, identifying key barriers and facilitators influencing its adoption within the specific ICU context using CFIR-informed qualitative methods. (3) Assess the impact of the implementation on nurses’ knowledge, behavior, and relevant organizational processes.

By concurrently evaluating patient outcomes and implementation factors, this study seeks to provide not only evidence for the efficacy of NLPW in the target setting but also actionable insights for its successful scaling and sustainability in similar contexts.

## Methods

### Design and context

Our study followed a mixed-methods design. As an Implementation study, the study findings will be reported following the Standards for Reporting Implementation Studies (StaRI) Statement [32] (Supplementary Material-checklist). The quantitative research utilized a historical control quasi-experimental design to verify changes in nurses’ behaviors, organizational environment, and patient-related outcome indicators before and after the evidence-based practice (EBP). The qualitative research employed semi-structured interviews to identify barriers and facilitators in the EBP implementation process.

The study was conducted in the Intensive Care Unit (ICU) of the Third Hospital of Peking University, Beijing, China. This hospital serves as a teaching hospital of Peking University. The ICU currently has 19 beds and more than 60 registered intensive care nurses. Approximately 1,000 critically ill patients are admitted annually, among whom over 500 receive IMV. The intervention was implemented from May to August 2024, while the control group data were collected from May to September 2023. The number of staff in the department has remained stable over the past two years, and before the study, the nurses had no prior experience with EBP.

### Ethical considerations

This study was approved by the Ethics Committee of Peking University Third Hospital (IRB00006761-M2023519).

### Inclusion and exclusion criteria

The study subjects comprised intensive care unit nurses and critically ill patients subjected to IMV.

#### Inclusion criteria for ICU nurses

Possession of a valid nursing qualification and employment as a clinical nurse in the target ICU.

#### Exclusion criteria for nurses

Nurses who were not directly involved in patient care during the study period.

#### Inclusion criteria for patients

1. Adult patients admitted to the Department of Critical Care Medicine at Peking University Third Hospital, who underwent endotracheal intubation and mechanical ventilation.
2. Age > 18 years
3. duration of mechanical ventilation < 7 days
4. provision of written informed consent by the patient or their legal caregiver

Voluntary participation in the study,

## Exclusion criteria for patients

1. patients with cervical spinal cord injuries
2. patients undergoing cardiopulmonary resuscitation (CPR)
3. comatose patients
4. patients with a history of chronic respiratory diseases

### Theoretical model

Implementation science provides essential frameworks and methodologies to systematically address this knowledge-to-practice gap [33]. It focuses not only on evaluating the effectiveness of an intervention but also on understanding how to successfully integrate evidence-based interventions into routine care within specific contexts, identifying barriers and facilitators, and developing strategies to promote sustainability [34]. Hybrid effectiveness-implementation designs (specifically Type 1) are increasingly recognized as efficient approaches, concurrently assessing clinical effectiveness while gathering crucial information on implementation feasibility and context [35–39].

The Consolidated Framework for Implementation Research (CFIR) [40] offers a comprehensive taxonomy of factors (intervention characteristics, outer and inner setting, individuals involved, implementation process) influencing implementation success, invaluable for diagnosing barriers and designing tailored strategies.

Theoretical frameworks are crucial for guiding rigorous implementation research. This study adopts the Knowledge to Action Framework (KTA) as the theoretical model [41]. Proposed in 2006 by Professor Graham’s research team in Canada, the KTA Framework aims to facilitate the translation of research findings into clinical practice by policymakers, administrators, and practitioners. The framework comprises two core components: “knowledge creation” and “knowledge application.”

The knowledge creation phase involves knowledge synthesis, integration, and generation to develop evidence-based tools tailored to clinical needs. The knowledge application phase guides the implementation of evidence in clinical settings through a structured process, including identifying problems, contextual adaptation, barrier assessment, implementation monitoring, outcome evaluation, and sustained application. This systematic approach ultimately enhances the quality of clinical care and improves patient health outcomes.

#### Nurse-led evidence-based protocolized weaning-establishment

A multidisciplinary research team was first established, comprising three PhDs in evidence-based medicine, four ICU nurses, and three ICU physicians.

During the knowledge generation phase, the research team conducted literature searches by the PICO framework and systematically retrieved evidence using the 6 S evidence pyramid model in a top-down approach, followed by quality assessment of the included evidence [42] (Supplementary Material S1). Ultimately, the study incorporated 3 clinical decision-making resources [43–45], 4 clinical guidelines [25–27], 2 evidence summaries [46, 47], and 1 systematic review [6] (Supplementary Material S2). The research team synthesized the best available evidence regarding protocolized weaning. The final NLPW program comprised four main domains and 23 specific recommendations within these domains (Supplementary Material S3). The four domains were: (1) Weaning Management, (2) Weaning Screening, (3) SBT Test, and (4) Extubation.

#### Nurse-led evidence-based protocolized weaning-application

This phase employed a stakeholder-centered approach, utilizing the CFIR as the theoretical foundation. Semi-structured interviews [40] (Supplementary Material S4) were conducted to qualitatively explore barriers and facilitators in EBP implementation across three dimensions: the external environment, internal environment, and individual characteristics. Data collection was performed in a quiet, enclosed room to minimize interruptions. Interviewers provided detailed explanations regarding the purpose and content of the interviews. All transcripts were anonymized and assigned unique identifiers within 24 h post-interview. Thematic analysis was performed using Colaizzi’s seven-step method until thematic saturation was achieved, after which no additional participants were recruited [48].

**Fig. 1:**
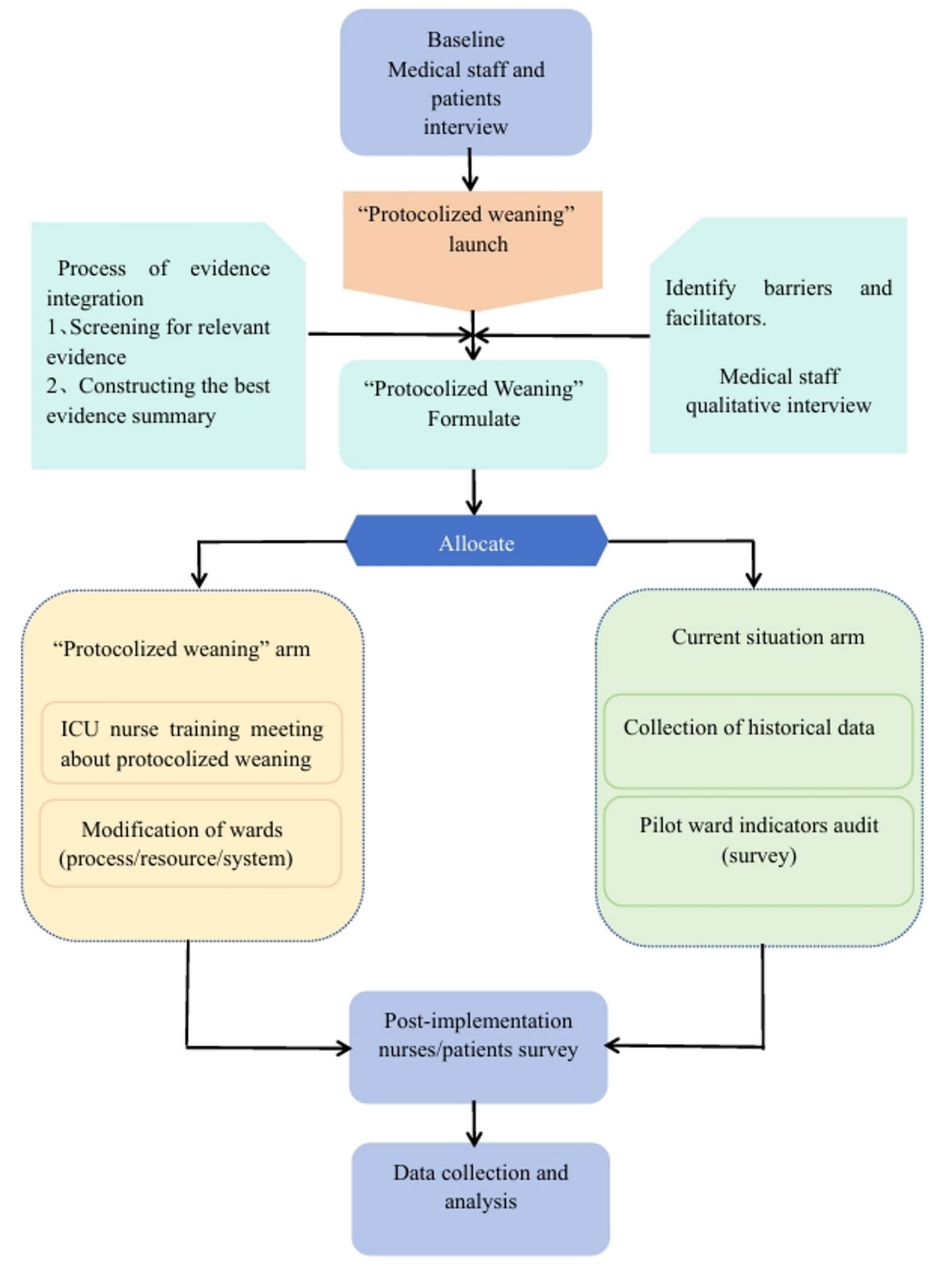
Nurse-led protocolized weaning implementation steps

Identified barriers and facilitators informed the development of a nurse-led, evidence-based action plan for protocolized weaning in mechanically ventilated patients. Based on the barrier analysis, a targeted training intervention was implemented for nurses in the ICU. Training consisted of three sessions delivered through a blended learning approach (online and in-person), covering: (1) enhancement of protocolized weaning expertise, (2) strategies for optimal EBP implementation, and (3) improvement in management skills for weaning mechanically ventilated patients. Unit managers were advised to refine workflows and optimize resource allocation in accordance with identified barriers to facilitate EBP adoption. The development and implementation steps of the action plan are illustrated in Fig. 1.

### Measurements

In this study, three categories of outcomes were evaluated:

1. Patient-related outcomes: changes in clinical indicators
2. Nurse-related outcomes: changes in knowledge levels
3. process evaluation: nurse adherence to best EBPs

#### Patient-related outcomes

The demographic data of patients were defined as clinical information obtained after hospital admission, with relevant data extracted from the electronic medical record system. The duration of mechanical ventilation was defined as the time from the initiation of the SBT to extubation. The weaning duration was calculated from the commencement of SBT until immediately before extubation. The length of ICU stay was defined as the hospitalization period from ICU admission to discharge. The number of reintubated patients within 24 h refers to those who required reintubation within 24 h after extubation.

#### Nurse-related outcomes

The professional knowledge and competency of nurses were assessed using a self-developed protocolized weaning knowledge test, which comprised 10 single-choice questions and 10 multiple-choice questions. Nurses in the pilot ward were tested before and after strategy implementation, with their knowledge levels determined based on scoring results. The barriers and facilitators of NLPW in clinical practice were identified.

#### Process assessment outcomes

The research team developed nine evaluation items based on previously established best clinical practices. Measurement outcomes were obtained through observation, review of medical records, and interviews with nurses. A higher number of nurses meeting the criteria for each review indicator corresponded to a higher implementation rate. The process evaluation items and methods are detailed in Table 1.

**Table 1:** Evaluation items for process assessment

No	Evaluation item
1	The department should have a nurse-led protocolized weaning plan and management process.
2	All mechanical ventilated patients underwent the SBT test once daily.
3	The sedation scores of patients undergoing protocolized weaning should be up to standard.
4	The nurses’ knowledge of protocolized weaning scheme questionnaire reached the standard of ≧ 80 points.
5	The department should have a nursing record sheet for protocolized weaning.
6	The department should have a promotion strategy for nurse-led protocolized weaning
7	Nurses had received training related to protocolized weaning in the past year, including weaning management, preparation for weaning, SBT test operation, and extubation.
8	The nurses were proficient in operating the protocolized weaning program.
9	The nurses were proficient in the use of ventilator mode.

###  Data collection

Clinical data from ICU patients who underwent endotracheal intubation and mechanical ventilation (control group) between May and September 2023 were collected by the research team via the hospital’s electronic medical record system. Pre- and post-intervention knowledge assessments of nurses were conducted through online questionnaires. Process evaluation outcomes in the pilot ward were retrospectively recorded before and after the implementation of the evidence-based practice project. Post-implementation data for the intervention group were collected concurrently during the implementation period (May-August 2024).

ICU nurses initiated daily pre-weaning assessments for mechanically ventilated patients at 6:00 AM. A pre-established NLPW flowchart was utilized for standardized patient evaluation during this preliminary phase. Upon successful completion of the weaning screening, nurses communicated the assessment results to the attending physician, who then issued orders regarding weaning initiation. Following physician directives, nurses transitioned the ventilator mode to Pressure Support Ventilation (PSV). After 30 min, the ventilator was discontinued and replaced with oxygen therapy via a tracheal catheter at a flow rate of 4–6 L/min for another 30 min. After protocolized weaning, nurses evaluated weaning outcomes and reported to the physician, who made the final determination regarding tracheal extubation. Subsequently, the research team collected and analyzed relevant clinical data from the patients. The NLPW flowchart is presented in supplementary material S5.

### Calculation of sample size

In this study, the duration of mechanical ventilation was adopted as the primary outcome measure. Based on previous research [49], protocolized weaning was estimated to reduce the mean duration of mechanical ventilation by 14 h. With an intervention-to-control group allocation ratio of 1:1, α set at 0.05, β at 0.20, and an anticipated dropout rate of 10%, a minimum of 147 participants per group was required.

### Statistical analysis

Statistical analysis was performed using SPSS 20.0 software. Continuous variables conforming to normal distribution were expressed as mean ± standard deviation or median and analyzed using t-tests. For non-normally distributed continuous variables, median and interquartile range were reported, and the Mann-Whitney U test was employed. Categorical variables were presented as frequencies and percentages, with comparisons made using χ² tests or Fisher’s exact probability test as appropriate. A P-value < 0.05 was considered statistically significant.

## Results

### Characteristics of study patients

**Fig. 2:**
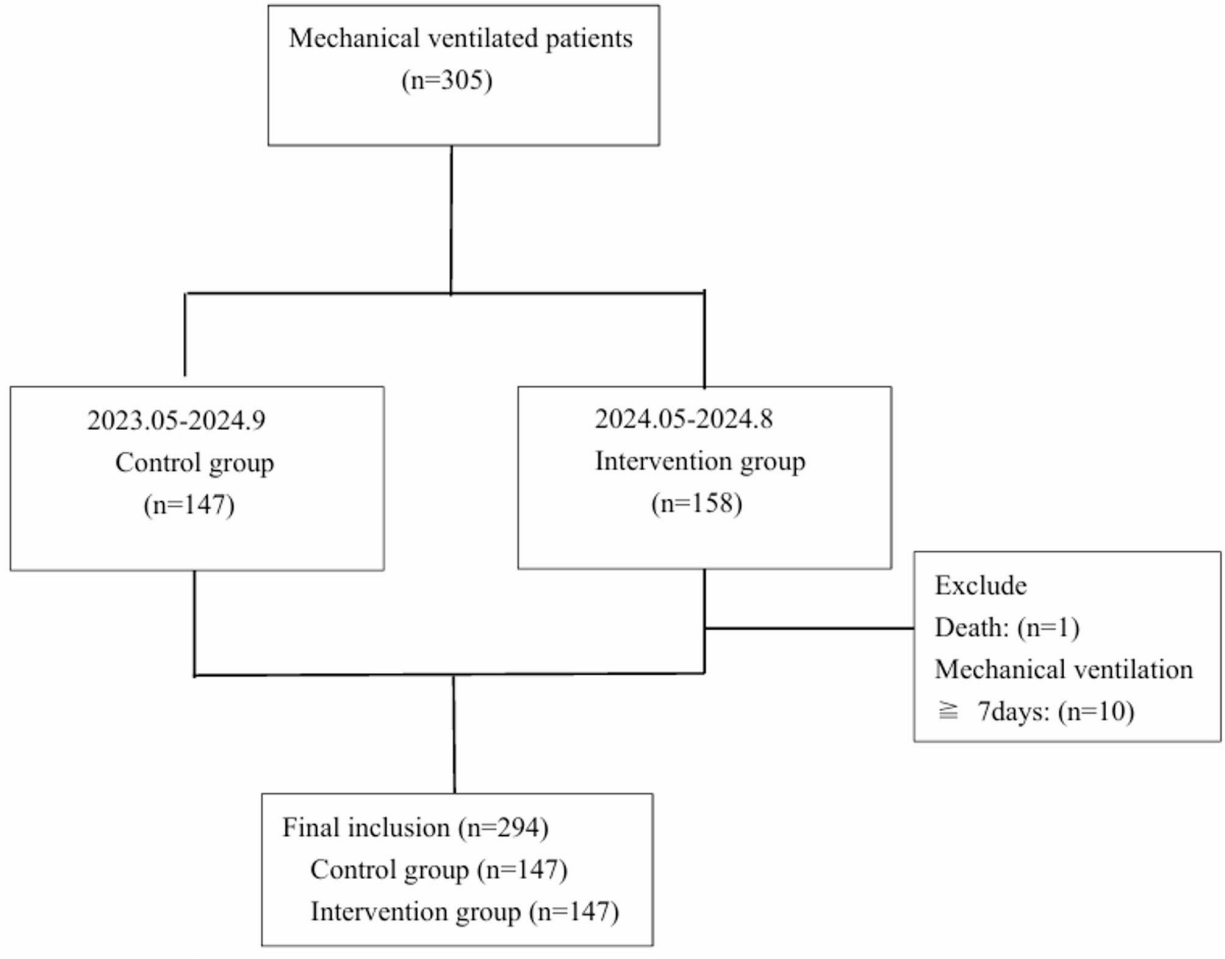
Flowchart of patients with mechanical ventilation included

A total of 305 critically ill patients were enrolled in this study, with 1 death and 10 cases requiring mechanical ventilation for over 7 days in the intervention group. Ultimately, 294 patients were included in the analysis: 147 in the intervention group and 147 in the control group (Fig. 2). The mean age was 62.32 ± 17.84 years, with males comprising 50.3% (*n* = 148) of the cohort. No statistically significant differences were observed between the two groups regarding age, sex, body mass index, patient origin, or ward source. The intervention group demonstrated significantly higher Acute Physiology and Chronic Health Evaluation II (APACHE-II) scores compared to the control group (95% CI: 1.29–3.14), indicating greater disease severity in the intervention group (Table 2).

**Table 2:** General information of patients in the two groups

		Intervention group (*n* = 147)	Control group (*n* = 147)	*P* value
Age (years)		61.86 ± 18.2	62.78 ± 17.5	0.66
Sex (male)		66(44.6)	82(55.4)	0.062
Body mass index > 28 (kg/m^2^)		23(52.3)	21(47.7)	0.744
APACHE-Ⅱ score		16.07 ± 3.8	13.85 ± 4.2	< 0.001
Source of patients				
	General ward (post-operation)	115(46.7)	131(53.3)	0.009
	Emergency ward	30(71.4)	12(28.6)	
	Transfer from other hospitals	2(33.3)	4(66.7)	
Source of wards				
	Cardiac surgery	1(50)	1(50)	0.158
	Orthopedics	30(71.4	12(28.6)	
	Neurosurgery	4(50)	4(50)	
	General surgery	52(46.4)	60(53.6)	
	Urinary surgery	11(32.4)	23(67.6)	
	Obstetrics	17(77.3)	5(22.7)	
	Vascular surgery	12(63.2)	7(36.8)	
	Intensive care unit	0	1(100)	
	Thoracic surgery	3(60)	2(40)	
	Gynecology	20(55.6)	16(44.4)	
	Plastic surgery	1(50)	1(50)	

### Outcomes of the patients

The duration of mechanical ventilation in the intervention group was shorter than that in the control group, but the difference was not statistically significant. The weaning duration in the intervention group was reduced by 1.2 h compared to the control group, with a statistically significant difference (*P* < 0.05). No significant differences were observed between the two groups in terms of ICU length of stay or the rate of reintubation within 24 h (Table 3).

**Table 3:** Clinical outcomes of the patients

Outcomes	Intervention group (*n* = 147)	Control group (*n* = 147)	*P* value
Duration of mechanical ventilation (hours)	20.41 ± 22.43	23.51 ± 29.13	0.308
Weaning duration (hours)	1.66 ± 0.92	2.8 ± 1.35	< 0.01
ICU length of stay (hours)	44.10 ± 37.05	56.00 ± 72.67	0.078
Reintubated at 24 h	1	0	0.316

*ICU* Intensive Care Unit

### Nurses-related outcomes

In this study, approximately 60 intensive care unit nurses participated in the professional knowledge assessment, including 58 before EBP implementation and 49 after EBP implementation. The general characteristics of the nurses are presented in Table 4. Following EBP implementation, the mean score on the nurse knowledge assessment increased from 70.34 ± 7.63 to 83.45 ± 5.08, demonstrating a statistically significant improvement (*P* < 0.05). Subgroup analysis based on Professional Title (junior vs. senior) showed that all experience levels demonstrated significant knowledge improvement, but junior nurses showed the greatest absolute improvement (from 65.3 ± 6.8 to 81.5 ± 4.2, *p* < 0.001) compared to senior nurses (from 75.6 ± 5.9 to 86.3 ± 4.5, *p* < 0.001). With no statistically significant difference in the magnitude of improvement between the two subgroups (*P* = 0.06).

**Table 4:** General information of nurses

		Before implementation (*n* = 58)	After implementation (*n* = 49)	*P* value
Gender	Male	23(39.7)	22(44.9)	0.584
Age		30.57 ± 5.66	31.57 ± 4.94	0.336
Level of education	Bachelor	40(69)	29(59.2)	0.292
	Junior college	18(31)	20(40.8)	
Professional Title	Senior	4(6.9)	4(8.2)	0.862
	Junior	41(70.7)	36(73.5)	
	Registered	13(22.4)	9(18.3)	
Job level	High (> 6 years experience)	17(29.3)	10(20.4)	0.379
	Middle (3–5 years experience)	29(50)	31(63.3)	
	Low (0–2 years experience)	12(20.7)	8(16.3)	
Specialty nurse	Yes	11(19)	7(14.3)	0.519
	No	47(81)	42(85.7)	

A total of nine healthcare professionals participated in the qualitative interviews, comprising four physicians and five nurses (Supplementary Material S6). Among the participants, one deputy department head and two head nurses took part in individual interviews.

Qualitative data analysis identified three main themes: (1) individual behavioral changes, (2) optimization of nursing workflows, and (3) utilization of resources to support clinical practice, along with nine corresponding sub-themes: attitude, idea, professional knowledge, belief, communication, lack of workflow, motivation, human resource, and laws and regulations (Table 5).

**Table 5:** Results of qualitative analysis

Themes	Sub themes	Facilitator	Barrier	CFIR domains
Individual behavioral changes	Attitude	Positive attitudes among medical staff		Characteristics of individuals
	Idea		Absence of relevant conceptual understanding	Characteristics of individuals
	Professional knowledge		Inadequate professional knowledge	Characteristics of individuals
	Belief	Potential benefits to patients		Outer setting
Optimize the nursing workflow	Communication		Poor communication between doctors and nurses	Characteristics of individuals
	Lack of workflow		Lack of standardized workflow	Inner setting
Utilization of resources to support clinical practice	Motivation	Appropriate incentive measures		Inner setting
	Human resource	Sufficient human resources and organizational support		Inner setting
	Laws and regulations		Insufficient regulatory and system guarantees	Outer setting

*CFIR* Consolidated Framework for Implementation Research

The results indicated that four facilitators promoted evidence-based nursing practice within the department: (1) potential benefits to patients, (2) appropriate incentive measures, (3) sufficient human resources and organizational support, and (4) positive attitudes among medical staff.

Conversely, five barriers were identified: (1) insufficient regulatory and system guarantees, (2) lack of standardized workflow, (3) absence of relevant conceptual understanding, (4) poor communication between doctors and nurses, and (5) inadequate professional knowledge.

### Process evaluation outcomes

**Fig. 3:**
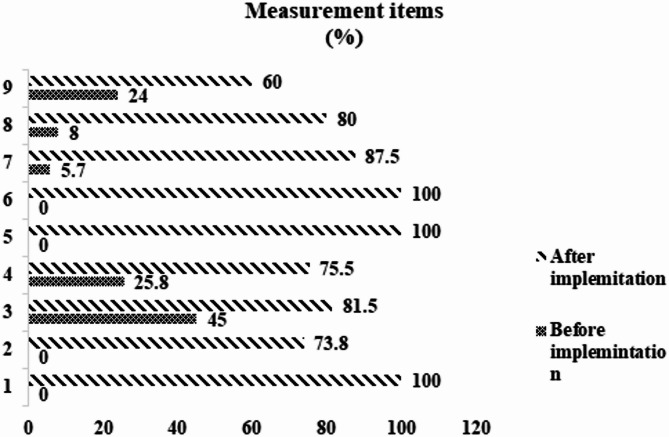
The implementation rate of 9 items

Following the implementation of EBP, the compliance rates for each measurement item were calculated. Among them, Item 1 (“The department shall establish a NLPW plan and management process”), Item 5 (“The department shall maintain protocolized weaning nursing records”), and Item 6 (“The department shall develop NLPW promotion strategies”) achieved 100% compliance rates, transitioning from nonexistent to fully implemented. Significant improvements were also observed in the compliance rates of other items (Fig. 3).

## Discussion

This hybrid type 1 effectiveness-implementation study demonstrates that a nurse-led evidence-based protocolized weaning (NLPW) program significantly reduced weaning duration by 1.2 h (*P* < 0.001) in critically ill patients undergoing IMV, without increasing 24-hour reintubation rates or ICU length of stay. Concurrently, the implementation process enhanced nurses’ knowledge of protocolized weaning by 13.1 points (*P* < 0.05) and optimized unit workflows through barrier-targeted interventions. These findings align with growing evidence supporting nurse-driven weaning protocols as safe and effective strategies for accelerating liberation from IMV, while providing novel insights into the contextual facilitators and barriers influencing sustainable implementation in resource-constrained settings.

The reduction in weaning duration (1.66 ± 0.92 vs. 2.8 ± 1.35 h) is clinically meaningful despite the absence of a significant decrease in total mechanical ventilation duration. This discrepancy may be attributed to several factors. First, the intervention group had higher baseline APACHE-II scores (16.07 ± 3.8 vs. 13.85 ± 4.2, *p* < 0.001), indicating greater disease severity, which could have necessitated longer periods of full ventilatory support prior to weaning readiness. Second, the cohort consisted predominantly of postoperative surgical patients with relatively short IMV durations (median ~ 1 day). In such a population, the absolute room for improvement in total IMV time is limited, and even a substantial reduction in weaning duration may not translate into a statistically detectable difference in overall ventilation hours due to ceiling effects [49]. Importantly, the protocol’s safety was affirmed by comparable reintubation rates (24 h) between groups, consistent with meta-analyses confirming NLPW does not increase extubation failure risk [6, 12]. The systematic morning assessment (initiated at 6:00 AM) facilitated earlier extubation (completed by 8:00 AM), improving patient comfort and aligning nursing workflows with circadian rhythms, a factor increasingly linked to weaning success [50]. This timing was chosen to align with standard ICU morning rounds and ensure assessments were completed before physician rounds.

The observed improvements—notably the 13.1-point increase in nurses’ knowledge scores (*P* < 0.05) and near-perfect compliance (>95%) with critical process indicators—demonstrate the efficacy of our theory-driven implementation approach. Qualitative insights revealed that success hinged on three interdependent factors: individual behavior change through targeted training empowered nurses to confidently lead SBT assessments, reducing reliance on physician availability; workflow optimization via standardized 6:00 AM assessments streamlined interdisciplinary coordination; and strategic resource utilization leveraged electronic health records for real-time auditing, enhancing accountability. These align with CFIR’s Inner Setting (workflow integration) and Characteristics of Individuals (skill-building) domains [51]. Crucially, high baseline nurse receptiveness to EBP (91.3%) signaled organizational readiness, providing fertile ground for protocol adoption—a contextual advantage often absent in failed implementations [52].

Our weaning duration reduction corroborates Roh et al.’s findings (1.9-hour reduction with nurse-led protocols) [53] but contrasts with Atefeh et al.’s reported 14-hour decrease in total IMV duration [49]. This divergence likely reflects differences in patient acuity: Atefeh’s cohort comprised medical ICU patients with longer ventilation needs (>5 days), whereas our surgical patients had shorter IMV exposure. The NLPW protocol mainly focuses on the nurses’ daily screening and leading implementation of the decision to remove the tube. This strategy significantly shortens the weaning duration of patients (the difference is clearly shown in Table 1), but has limited effect on short-term mechanical ventilation patients in terms of shortening the duration of mechanical ventilation. Our study extends prior work by integrating implementation science frameworks (KTA, CFIR) to systematically address context-specific barriers, a gap noted in recent reviews of weaning protocols [54, 55].

This study highlights the critical value of the nurse-led weaning protocol (NLPW) in addressing prevalent challenges in ICU. By standardizing nursing procedures to reduce practice variation [13], NLPW effectively shortens the duration of patient weaning. Successful implementation requires multifaceted strategies: structured competency-based training on weaning criteria and SBT execution; deliberate workflow integration, such as establishing dedicated morning assessment sessions; and fostering interprofessional trust through shared protocols to ensure physician endorsement of nurse-led decision-making [12].

The findings hold particular relevance for critical care delivery in developing regions where resource limitations exacerbate challenges in ventilator weaning [56]. First, NLPW directly addresses the critical shortage of respiratory therapists prevalent in these settings [57] by leveraging existing nursing cadres—a workforce typically more abundant and continuously available at the bedside [58–60]. Second, the protocol’s emphasis on structured checklists and objective criteria mitigates reliance on subjective physician judgments, which may be inconsistent in overburdened healthcare systems [61]. Third, the implementation strategy’s focus on workflow integration and task-shifting offers a scalable model to improve care standardization without requiring substantial new infrastructure investments [62].

However, successful adaptation necessitates contextual modifications: training must account for variable nurse-to-patient ratios and education levels, while protocol complexity should align with available monitoring resources [63]. Policymakers in these regions should prioritize NLPW as a cost-effective strategy to reduce ICU length of stay—a key driver of financial toxicity in low-resource contexts [64]. The integration of NLPW into clinical practice frameworks should be emphasized, particularly in areas with a shortage of respiratory therapists [65]. This approach would formalize the pivotal role of nurses in ventilator weaning and standardize evidence-based care pathways across diverse healthcare settings [66].

## Limitations

Several limitations warrant consideration. First, the single-center ICU setting restricts generalizability to resource-constrained environments, and the barriers and facilitators were identified solely within one tertiary hospital in Beijing, limiting transferability to institutions with differing organizational cultures or resource profiles. Second, the use of historical controls introduces potential confounding from unmeasured temporal factors, though the intervention group’s higher baseline APACHE-II scores suggest robustness against selection bias. Third, short-term follow-up precludes assessment of long-term protocol sustainability or lasting behavioral changes among nurses.

## Conclusion

This study confirms that a systematically implemented, NLPW program safely accelerates liberation from mechanical ventilation in ICU patients while enhancing nursing expertise and unit efficiency. Successful adoption hinges on addressing contextual barriers through tailored training, workflow redesign, and resource optimization. Future efforts should focus on scaling NLPW across diverse ICU settings and evaluating its impact on long-term clinical and operational outcomes. Multicenter randomized designs with cost-effectiveness analyses are needed to further validate these findings across diverse ICU contexts.

## Supplementary Information

Supplementary Material 1.

Supplementary Material 2.

## Data Availability

The data that support the findings of this research are available from the corresponding author upon reasonable request.
