# Supplementary material for: Nurse-led evidence-based protocolized weaning for invasive mechanical ventilation patients in the ICU: a hybrid type 1 effectiveness-implementation study
Source: BMC Pulm Med. 2025 Oct 27;25:491. doi: 10.1186/s12890-025-03967-5 (PMC12557896; doi:10.1186/s12890-025-03967-5)
Supplement: Supplementary file 2 — Supplementary Material 2. [file 12890_2025_3967_MOESM2_ESM.docx]

**Supplementary Material**

**S1: Search strategy and literature screening process.**

We systematically searched Chinese and English databases, professional websites, and guideline repositories. The databases included the JBI Database, PubMed, Embase, and CINAHL. Professional websites comprised those maintained by the American Thoracic Society, American Association for Respiratory Care, and American Association of Critical-Care Nurses. Guideline sources included the Guidelines International Network and Scottish Intercollegiate Guidelines Network. A search strategy was constructed using PubMed as an example. The search period spanned from each database's inception to October 2022.

＃1（intensive care unit）OR（intensive care OR intensive）

＃2（invasive mechanical ventilation）OR（invasive ventilation）

＃3（mechanical ventilation weaning）OR（ventilation weaning）OR（ventilator weaning）

＃4（weaning protocol）OR （liberation from mechanical ventilation） OR （discontinuing from mechanical ventilation）OR（readiness testing） OR（spontaneous breathing trial）

＃5（practice guideline） OR （guideline）OR（evidence）

＃6（child） OR （pediatric） OR （newborn）

＃7 ＃1 AND ＃2 AND ＃3 AND ＃4 AND ＃5 NOT ＃6


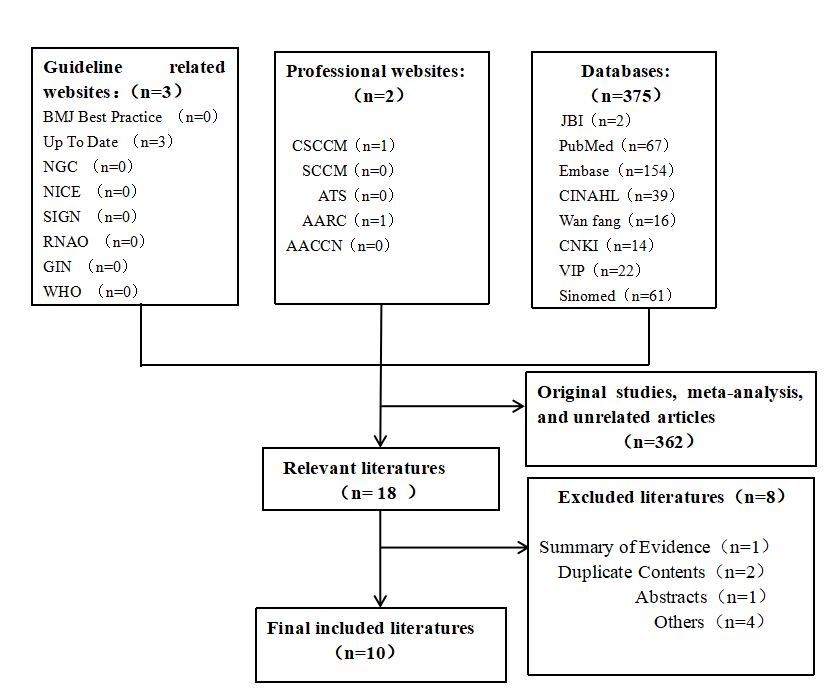


**S2: Search result**

| **No** | **Included literatures** | **Literature resources** | **Publish**  **time** | **Type of literature** | **Subject content** |
| --- | --- | --- | --- | --- | --- |
| 1 | Epstein | UpToDate | 2022 | Clinical decision | Weaning from mechanical ventilation: Readiness testing |
| 2 | Epstein | UpToDate | 2022 | Clinical decision | Initial weaning strategy in mechanically ventilated adults |
| 3 | Hyzy | UpToDate | 2022 | Clinical decision | Extubation management in the adult intensive care unit |
| 4 | TimothyD | PubMed | 2017 | Guideline | Physicians Clinical Practice Guideline: Liberation from Mechanical  Ventilation in Critically Ill Adults |
| 5 | GregoryA | PubMed | 2017 | Guideline | Official Executive Summary Physicians Clinical Practice Guideline: Liberation from Mechanical Ventilation in Critically Ill Adults |
| 6 | E.Wesley | PubMed | 2001 | Guideline | Mechanical Ventilator Weaning Protocols Driven by Nonphysician Health-Care Professionals |
| 7 | MacIntyre | AARC | 2002 | Guideline | Evidence-Based Guidelines for Weaning and Discontinuing Ventilatory Support |
| 8 | Vivek | JBI | 2021 | Summary Evidence | Endotracheal tube: extubation |
| 9 | Cynthia | JBI | 2021 | Summary Evidence | Mechanical ventilation: weaning interventions in critical ill adults |
| 10 | Hirzallah | PubMed | 2019 | Systematic review | Nurse-led weaning protocol for mechanically ventilated adult patients |

**S3: N**urse-led protocolized weaning program contents list

| **Category** | **Items** | **Recommendation [Evidence Type, Issuing Institution, Publication Time (Recommendation Strength/Evidence Level)]** |
| --- | --- | --- |
| Weaning Management | （1-4） | 1. Patients on mechanical ventilation for >24 hours should adopt a protocolized weaning strategy. [CD, Epstein1, 2022(-/4)] |
|  |  | 2. Programmed weaning protocols should be developed for non-physician healthcare professionals. [CPG, AHCPR, 2001(√/3)] |
|  |  | 3. Protocolized weaning reduces mechanical ventilation time in general medical and surgical ICUs, except for neurosurgical ICUs. [SR, Hirzallah, 2019(-/2)] |
|  |  | 4. For implementing programmed weaning protocols, consider the following strategies: multidisciplinary teams, evidence-based goal-setting, and effective behavior change strategies (interactive education, leadership opinion reminders, auditing, feedback). [CPG, AHCPR, 2001(-/5)] |
| Weaning Screening | （5-8） | 5. Patients on mechanical ventilation for >24 hours are recommended to adopt a minimal sedation strategy. [CPG, ACCP, 2016(-/4)] |
|  |  | 6. Patients on mechanical ventilation for >24 hours should undergo daily assessment of weaning readiness. [CPG, AHCPR, 2001(√/1)] |
|  |  | 7. For patients on mechanical ventilation for >24 hours who fail to wean, the causes of failure should be identified as soon as possible. [CPG, ACCP, 2002(-/3)] |
|  |  | 8. Objective criteria for weaning assessment: ① The cause of respiratory failure has reversed; ② Sufficient oxygenation (PaO2/FIO2 >150-200 or PaO2/FIO2 ≥150; PEEP ≤5-8 cmH2O or PEEP ≤5 cmH2O; FIO2 ≤0.4-0.5 or FIO2 ≤0.4; SpO2 ≥90%) without acidosis (pH ≥7.25); ③ Hemodynamic stability with heart rate <140 beats/min, no active myocardial ischemia, hypotension, or need for low-dose dopamine <5 μg/kg/min; ④ Awake with GCS ≥13 points, no continuous sedative infusion; ⑤ Ability for spontaneous breathing. [CPG, ACCP, 2002(-/2)] |
| SBT Test | （9-15） | 9. For patients who pass the weaning screening, use SBT rather than progressive PSV or IMV for weaning. [CD, Epstein1, 2022(√/2)] |
|  |  | 10. After patients pass the SBT test, physicians should consider timely extubation. [CPG, AHCPR, 2001(√/3)] |
|  |  | 11. When the SBT test fails, consider remedial measures: select a safe and comfortable ventilation mode, and elevate the head of the bed by 30-45 degrees. [CD, Epstein2, 2022(-/5)] |
|  |  | 12. For patients with failed SBT, the causes should be identified. Once the causes are corrected, SBT should be repeated every 24 hours. [CPG, ACCP, 2002(-/2)] |
|  |  | 13. SBT should use low-level pressure support of 5-8 cmH2O rather than T-tube or CPAP. [ES, JBI, 2021(√/1)] |
|  |  | 14. Each SBT should last for 30 minutes. [CPG, ACCP, 2002(-/2)] |
|  |  | 15. Objective criteria for SBT failure: Respiratory: respiratory rate ≥35 breaths/min, accessory muscle use; Hemodynamic: heart rate >140 beats/min or change >20%, heart rate <50 beats/min, systolic blood pressure <90 mmHg or >180 mmHg; Gas exchange: SpO2 <90%, PaO2 <50 mmHg; Diaphoresis; Consciousness: decreased level of consciousness, somnolence, anxiety, delirium. [CD, Epstein2, 2022(-/4)] |
| Extubation | （16-23） | 16. For patients at high risk of laryngeal edema (prolonged intubation ≥36 hours to ≥6 days, age >80 years, large-caliber ETT >8 mm for males, >7 mm for females, intubation trauma, history of asthma, aspiration), perform a cuff leak test before extubation. [CPG, ATS, 2016(√/3)] |
|  |  | 17. For patients who successfully pass the SBT, extubation should be performed based on assessment of airway patency and airway protection ability. [CPG, ACCP, 2002(-/4)] |
|  |  | 18. For patients who pass the SBT but are at high risk of extubation failure (elderly, COPD, congestive heart failure, hypercapnia), non-invasive sequential ventilation therapy is recommended after extubation. |
|  |  | 19. Patients with weak cough ability and GCS <8 points should have extubation delayed. [ES, JBI, 2021(-/1)] |
|  |  | 20. For patients at high risk of laryngeal edema with a negative cuff leak test, the decision to extubate should be made based on specific conditions. [ES, JBI, 2021(-/2)] |
|  |  | 21. For patients with a positive cuff leak test who require extubation, administer glucocorticoids 4 hours before extubation. [CPG, ATS, 2016(-/3)] |
|  |  | 22. Generally, suspend nasogastric feeding for 1 hour before extubation. [CPG, ACCP, 2016(-/5)] |
|  |  | 23. After extubation, close monitoring should be performed to prevent reintubation: sputum suction, bronchodilators, diuretics, non-invasive positive pressure ventilation. [ES, JBI, 2021(-/2)] |

**S4: Qualitative interview outline**

| **CFIR domains** | **Questions for Interview** |
| --- | --- |
| **External environment**  Interview questions were determined based on patient needs, cooperation, policies, and other factors | 1. In view of the needs of patients, what effect do you think this intervention will have? 2. Does your department communicate much with other colleagues in this field? 3. Do you think there are any regulations that will affect the implementation of nurse-led procedural weaning? |
| **Internal environment**  Interview questions were set according to implementation readiness, resources, organizational incentives, goals and other factors | 1. What support do you want from your organization's leaders to help achieve success? 2. Is the implementation of this measure consistent with your existing workflow and practices? 3. Does your department have the resources (equipment, personnel) to implement this initiative? 4. What kind of incentives do you think will help to make this successful? |
| **Individual characteristics**  Interview questions were determined according to nurses' attitude, cognition, confidence and organizational identity towards the implementation of the intervention | 1. How much do you know about protocolized weaning? 2. How do you think about the cooperation of the staff in the department in routine? 3. How confident are you in successfully implementing protocolized weaning of the patient？ |

**S5: Nurse-led protocolized weaning flowchart**

**
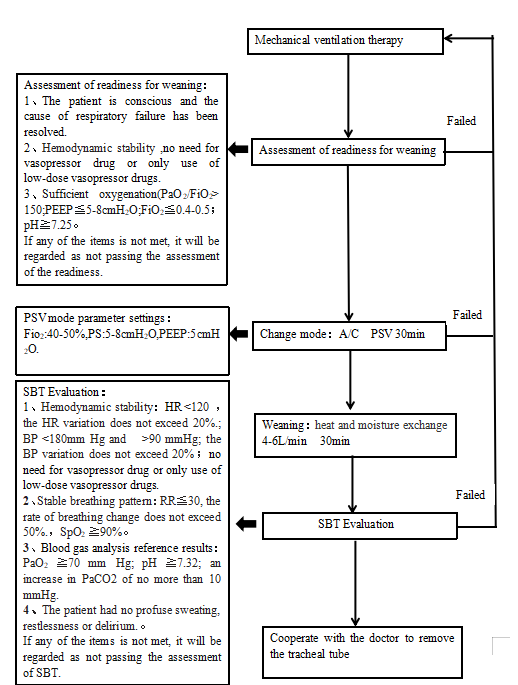
**

**S6: General information of qualitative interview participants**

|  | **Median(n)** | **IQR** |
| --- | --- | --- |
| Age | 35 | 25-48 |
| Work experience in ICU(years) | 10 | 4-22 |
|  | **Number(n)** | **Percentage(%)** |
| Sex |  |  |
| Female | 5 | 55.6 |
| Male | 4 | 44.4 |
| Employment classification |  |  |
| Registered Nurses | 5 | 55.6 |
| Physicians | 4 | 44.4 |
| level of education |  |  |
| College degree | 3 | 33.4 |
| Bachelor degree | 2 | 22.2 |
| Master | 1 | 11.0 |
| Doctor | 3 | 33.4 |

IQR: Interquartile range
